# Salvianolic Acid Exerts Cardioprotection through Promoting Angiogenesis in Animal Models of Acute Myocardial Infarction: Preclinical Evidence

**DOI:** 10.1155/2017/8192383

**Published:** 2017-06-21

**Authors:** Long-jie Yu, Ke-Jian Zhang, Jia-Zhen Zhu, Qun Zheng, Xiao-Yi Bao, Saroj Thapa, Yan Wang, Mao-Ping Chu

**Affiliations:** ^1^Department of Cardiology, The Second Affiliated Hospital and Yuying Children's Hospital of Wenzhou Medical University, Wenzhou 32500, China; ^2^Children's Heart Center, The Second Affiliated Hospital and Yuying Children's Hospital of Wenzhou Medical University, Institute of Cardiovascular Development and Translational Medicine, Wenzhou Medical University, Wenzhou 325000, China

## Abstract

Radix *Salviae miltiorrhizae*, danshen root (danshen), is one of the widely used Chinese herbal medicines in clinics, containing rich phenolic compounds. Salvianolic acid is the main active compound responsible for the pharmacologic effects of danshen. Here, we aimed to evaluate the effects of salvianolic acid on cardioprotection through promoting angiogenesis in experimental myocardial infarction. Studies of salvianolic acid in animal models of myocardial infarction were obtained from 6 databases until April 2016. The outcome measures were vascular endothelium growth factor (VEGF), blood vessel density (BVD), and myocardial infarct size. All the data were analyzed using Rev-Man 5.3 software. Ultimately, 14 studies were identified involving 226 animals. The quality score of studies ranged from 3 to 6. The meta-analysis of six studies showed significant effects of salvianolic acid on increasing VEGF expression compared with the control group (*P* < 0.01). The meta-analysis of the two salvianolic acid A studies and three salvianolic acid B studies showed significantly improving BVD compared with the control group (*P* < 0.01). The meta-analysis of five studies showed significant effects of salvianolic acid for decreasing myocardial infarct size compared with the control group (*P* < 0.01). In conclusion, these findings demonstrated that salvianolic acid can exert cardioprotection through promoting angiogenesis in animal models of myocardial infarction.

## 1. Introduction

Ischemic heart disease (IHD) remains the leading cause of death worldwide [1]. According to the World Health Organization report, 740 million people die of IHD annually all around the world, accounting for the death of 13.2% of the total population [2]. Myocardial infarction (MI) is one of the main manifestation of IHD, which makes myocardial necrosis or apoptosis in a short time [3], leading to heart failure with a poor prognosis [4]. It has been ranked as the leading cause of death in IHD [5].

In recent years, there are many types of treatments for MI, such as reducing incidence of coronary atherosclerosis [6], antithrombotic therapy including vitamin K antagonists [7], antiplatelet therapy with low-dose aspirin [8], and clopidogrel [9]. In addition, invasive vascular reconstruction is widely used, which improves coronary perfusion, such as percutaneous coronary intervention (PCI) and coronary artery bypass grafting (CABG) [10]. In the short term, clinical interventions and treatments of MI have achieved positive effectiveness [11]. However, the side effect of drugs such as lipid-lowering drugs leading to skeletal muscle, metabolic and neurological adverse events [12], antithrombotic therapy [13] and/or anti-platelet therapy [14] leading to bleeding, and the high incidence rate of restenosis or stent thrombosis limits the long-term success of treatment [15]. Thus, the promotion of therapeutic angiogenesis as a new treatment strategy has been proposed. Angiogenesis appears in all vascularized organs during the whole embryonic development stage, formatting of new blood vessels from pre-existing ones [16]. Although ischemia leads to endogenous myocardial angiogenesis, it cannot reach the effect to maintain normal capillary density [17]. Therefore, therapeutic stimulation of angiogenesis has been regarded as an effective treatment for myocardial ischemia [18].

Radix *Salviae miltiorrhizae*, danshen root (danshen), the dried root of *Salvia miltiorrhiza* Bge., known as a popular traditional Chinese herbal medicine, has been widely used and well received for the treatment of coronary artery diseases, such as angina pectoris and MI [19]. Salvianolic acid is the main active compound responsible for the pharmacologic effects of danshen [20] and exerts the significant cardiovascular protection [21]. Currently, various studies have indicated its significant function of promoting angiogenesis [22].

The use of preclinical systematic review can more systematically evaluate the efficacy, identify an area for testing in further animal experiments, provide reliable information about the drugs study, and list the base of future clinical research [23]. However, currently, there is no systematic review in this area. Thus, the aim of this study is to evaluate the effects of salvianolic acid on cardioprotection through promoting angiogenesis in animal experiments of MI.

## 2. Methods

### 2.1. Search Strategies

We searched studies of salvianolic acid in animal models of acute myocardial infarction from PubMed, EMBASE, Chinese National Knowledge Infrastructure (CNKI), VIP information database, and Wanfang Data information site from inception to April 2016. The search term used was “danshen OR *Salvia miltiorrhiza* OR Salvianolic acid OR Daiclzein” AND “myocardial infarction OR Myocardial Ischemia OR myocardialischemia OR myocardial infarct OR myocardial stems.” All the research objects were limited to animals.

### 2.2. Inclusion/Exclusion Criteria

We included studies about the effect of salvianolic acid on animal models with myocardial infarction, in which the outcome measures were vascular endothelium growth factor (VEGF) and/or blood vessel density (BVD). To prevent bias, inclusion criteria were prespecified as follows: (1) acute myocardial infarction (AMI) experimental model was induced by ligating of the left anterior descending coronary artery (LAD); (2) experimental drug was Salvianolic acid; and VEGF and/or BVD (3) is the primary outcome measurement and (4) is compared with control animal models receiving saline or no treatment. Prespecified exclusion criteria were treatment with single danshen or danshen-based prescription, a nonmyocardial infarct model, no control group, and duplicate publications.

### 2.3. Data Extraction

Two authors independently extracted data as follows: (1) publication year and the first author's name; (2) the information of experimental animals including number, species, sex, weight, and age; (3) a model of myocardial infarction; (4) the time of giving experimental drug; (5) the type and the administration methods of anesthetic; (6) the characteristics of treatment used in the experimental group containing the types of salvianolic acid, administration method, and duration of treatment; (7) the primary outcome measures, other outcome measures, and timing for outcome assessments; and (8) side effect. If there were many different time point outcomes, only the last was recorded. Likewise, if the experimental animals received different doses of the drug, only the highest dose was recorded. If the primary data were incomplete, further information was retrieved by contacting with authors. For each comparison, we extracted the mean value and standard deviation from each experimental and control group of every study. Discrepancies were resolved after discussion between the two authors.

### 2.4. Quality Assessment

We evaluated the methodological quality of the included studies using the ten-item scale [24] with minor modification as follows: (a) peer-reviewed publication; (b) control of temperature; (c) random allocation to treatment or control; (d) blinded induction of model; (e) blinded assessment of outcome; (f) use of anesthetic without significant intrinsic vascular protection activity; (g) appropriate animal model (aged, diabetic, or hypertensive); (h) sample size calculation; (i) compliance with animal welfare regulations; and (j) statement of potential conflict of interests. Every item was given one point. Two authors independently evaluated the study quality, and the final result was identified by discussion when countering disagreement.

### 2.5. Statistical Analysis

All the data of VEGF and BVD were considered as continuous data, and then, we used the standard mean difference (SMD) with the random effect model to assess the comprehensive results, because of the heterogeneity between multistudies. Then we utilized *I*^2^ statistic to estimate heterogeneity. The significance of differences between *n* groups was estimated by partitioning heterogeneity and by using the *χ*2 distribution with *n*−1 degrees of freedom (df), where *n* equals the number of groups. The publication bias was expressed by a funnel plot. Probability values of 0.05 were considered significant. We utilized RevMan version 5.3 to carry out the meta-analysis.

## 3. Results

### 3.1. Study Inclusion

**Figure 1:**
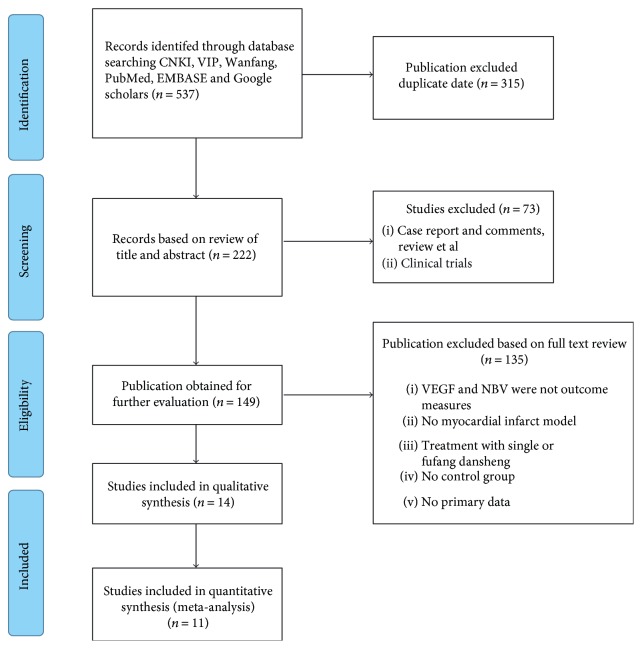
Flow diagram.

We searched 573 potentially relevant studies, and 315 were excluded because of duplication. After screening titles and abstracts, 73 studies were excluded because of a nonanimal study, clinical trial, case report, comments, or review. By reading the full text of the remaining articles, 135 studies were excluded because of at least one of the following reasons: (1) the outcome measures did not include VEGF and/or BVD; (2) nonmyocardial infarct model; (3) treatment with single danshen or danshen-based prescriptions; (4) no control group; and (5) duplicate publications. Ultimately, 14 eligible studies were included in qualitative synthesis and 11 eligible studies [25–38] in quantitative synthesis (Figure 1).

### 3.2. Study Characteristics

Fourteen studies with 226 animals were included. All the studies were published between 2004 and 2016, including four studies [25–27, 38] in English and seven studies [29, 32–37], two online PhD theses [30, 31], and one online Master's thesis [28] in Chinese. A male/female Sprague Dawley rat, male Wistar rat, and male piglet model were used in 12 studies [25–31, 33, 34, 36–38], 1 study [35], and 1 study [32], respectively. All the myocardial infarction models were produced by ligation of the LAD. Twelve studies used blood vessel density (BVD) [26–35, 37, 38], ten studies [25–29, 31, 33, 34, 36, 38] used VEGF, and eight studies [25–27, 30, 31, 34, 35, 37] utilized myocardial infarct size as outcome measures. Anesthetic was reported in 13 studies, including pentobarbital (*n* = 3) [30, 34, 35], urethane (*n* = 2) [26, 28], chloral hydrate (*n* = 4) [25, 29, 31, 33], ether (*n* = 2) [36, 37], ketamine and diazepam (*n* = 1) [32], and hydrochloride (*n* = 1) [27], whereas one study [38] did not report the anesthetic. Seven studies [25, 26, 30, 34, 35, 37, 38] used salvianolic acid B, two studies [27, 31] used salvianolic acid A, and five studies [28, 29, 32, 33, 36] used mixed salvianolic acids. There were three administration methods, including intragastric administration (*n* = 7) [26, 28, 30, 33, 35–37], intravenous administration (*n* = 5) [25, 27, 31, 32, 34], and intraperitoneal administration (*n* = 1) [29]. The characteristics of the included studies are concluded in Table 1.

### 3.3. Study Quality

The quality score of studies ranged from 3 to 6. All studies were publications in a peer-reviewed journal or thesis. Five studies reported control of room temperature. All studies described random allocation to the groups. Thirteen studies used anesthetic without significant intrinsic vascular protection activity. No studies described a sample size calculation. Four studies reported a compliance with animal welfare regulations, and five studies mentioned a statement of potential conflict of interests. None of the studies described masked induction of appropriate animal models (aged, diabetic, or hypertensive). The methodological quality is concluded in Table 2.

### 3.4. Effectiveness

#### 3.4.1. VEGF

**Figure 2:**
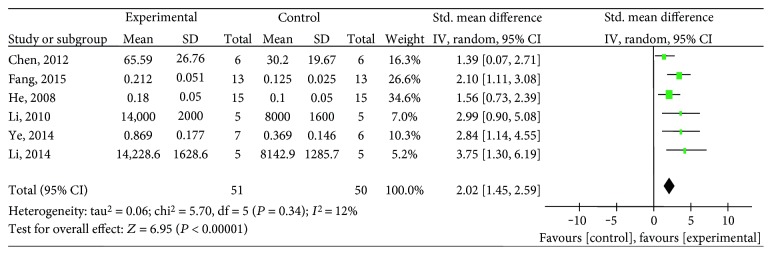
The forest plot: effects of salvianolic acid for increasing VEGF expression compared with the control group.

**Figure 3:**
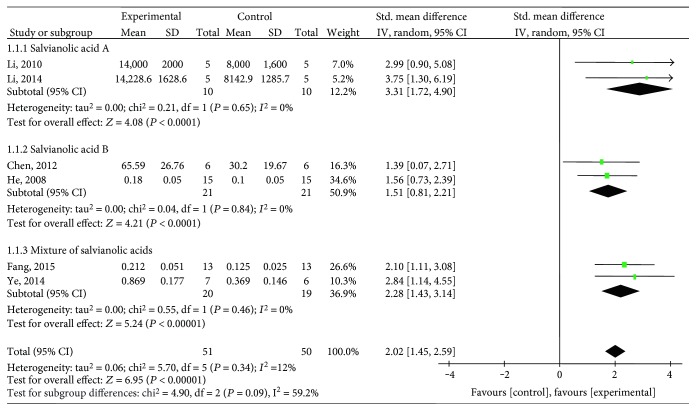
The forest plot: subgroup analysis of salvianolic acid A, salvianolic acid B, and a mixture of salvianolic acids for improving VEGF compared with the control group.

**Figure 4:**
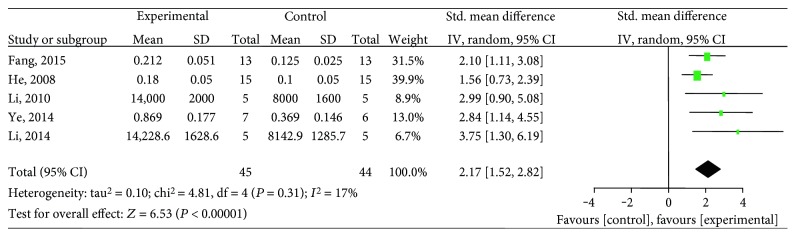
The forest plot: effects of salvianolic acid in male animals for increasing VEGF expression compared with the control group.

Ten studies [25–29, 31, 33, 34, 36, 38] utilized VEGF as the outcome measure. The meta-analysis of six studies [26, 27, 29, 31, 34, 36] showed significant effects of salvianolic acid for increasing VEGF expression compared with the control group (*n* = 101, SMD 2.02, 95% CI: 1.45∼2.59, *P* < 0.00001; heterogeneity *χ*2 = 5.70, *P* = 0.34, *I*^2^ = 12%), Figure 2. The subgroup analysis showed that salvianolic acid A [27, 31] (*n* = 20, SMD 3.31, 95% CI: 1.72∼4.90, *P* < 0.0001; heterogeneity *χ*2 = 0.21, *P* = 0.65, *I*^2^ = 0%), salvianolic acid B [26, 34] (*n* = 42, SMD 1.51, 95% CI: 0.81∼2.21, *P* < 0.0001; heterogeneity *χ*2 = 0.04, *P* = 0.84, *I*^2^ = 0%), and a mixture of salvianolic acids [29, 36] (*n* = 39, SMD 2.28, 95% CI: 1.43 ∼ 3.14, *P* < 0.00001; heterogeneity *χ*2 = 0.55, *P* = 0.46, *I*^2^ = 0%) were significantly increasing VEGF expression compared with the control group, respectively (Figure 3). After removing one study [34] that used female animals, the meta-analysis of five studies [26, 27, 29, 31, 36] that used male animals showed significantly increasing VEGF expression compared with the control group (*n* = 89, SMD 2.17, 95% CI: 1.52∼2.82, *P* < 0.00001; heterogeneity *χ*2 = 4.81, *P* = 0.31, *I*^2^ = 17%), Figure 4. The remaining 4 studies [25, 28, 33, 38] failed to pool the analysis because of the absence of primary data, but all of them reported significant effects of salvianolic acid for increasing VEGF expression compared with the control group (*P* < 0.05 or *P* < 0.01).

#### 3.4.2. BVD

**Figure 5:**
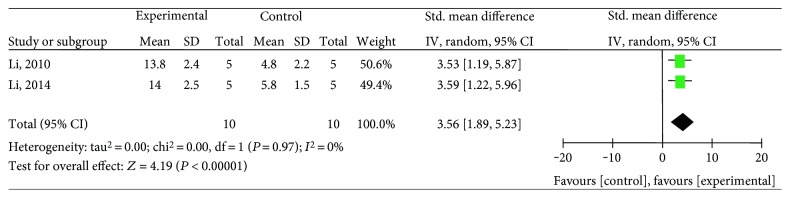
The forest plot: effects of salvianolic acid A for improving BVD compared with the control group.

**Figure 6:**
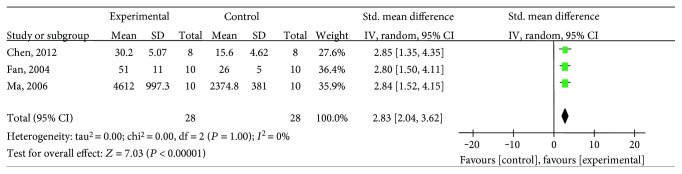
The forest plot: effects of salvianolic acid B that used pentobarbital as anesthetic for improving BVD compared with the control group.

Twelve studies [26–35, 37, 38] utilized BVD as the outcome measure, including salvianolic acid A [27, 31], salvianolic acid B [26, 30, 34, 35, 37, 38], and salvianolic acid mixture [28, 29, 32, 33]. The meta-analysis of the two salvianolic acid A studies [27, 31] showed significantly improving BVD compared with the control group (*n* = 20, SMD 3.56, 95% CI: 1.89∼5.23, *P* < 0.00001; heterogeneity *χ*2 = 0, *P* = 0.97, *I*^2^ = 0%), Figure 5. The meta-analysis of the five salvianolic acid B studies [26, 30, 34, 35, 37] showed significantly improving BVD compared with the control group (*n* = 107, SMD 1.9, 95% CI: 0.9 ∼ 2.9, *P* = 0.0002; heterogeneity *χ*2 = 16.47, *P* = 0.002, *I*^2^ = 76%). Owing to the significant statistical heterogeneity, we utilized subgroup analyses to explore the sources of the heterogeneity. Among the five included studies, three studies [30, 34, 35] used pentobarbital as anesthetic, one study [26] used urethane, and the last one [37] used ether. The meta-analysis of three studies [30, 34, 35] showed significant effects of salvianolic acid B for improving BVD compared with the control group (*n* = 56, SMD 2.83, 95% CI: 2.04∼3.62, *P* < 0.00001; heterogeneity *χ*2 = 0, *P* = 1, *I*^2^ = 0%), Figure 6, suggesting that anesthetic was the potential cause of the heterogeneity. Additionally, after removing two studies [34, 35] which used female animals, the meta-analysis of three studies [26, 30, 37] showed significant effects of salvianolic acid B for improving BVD compared with the control group (*n* = 71, SMD 1.38, 95% CI:0.26∼2.49, *P* = 0.02; heterogeneity *χ*2 = 8.04, *P* = 0.02, *I*^2^ = 75%). The reason of the high heterogeneity was possibly different anesthetics used. The remaining one salvianolic acid B study [38] failed to analyze because of the absence of primary data, but it also reported significant increasing BVD compared with the control group (*P* < 0.05). The meta-analysis of the 3 mixtures of salvianolic acid studies [29, 32, 33] showed significant effects for improving BVD compared with the control group (*n* = 54, SMD 8.46, 95% CI: 1.40∼15.53, *P* < 0.0001; heterogeneity *χ*2 = 19.78, *P* = 0.02, *I*^2^ = 90%). The reason of the high heterogeneity might be that each of them had different types of salvianolic acid. The remaining one mixture of a salvianolic acid study [28] failed to pool the analysis because of the absence of primary data. However, all of them reported a positive effect on increasing BVD compared with the control group (*P* < 0.05 or *P* < 0.01).

#### 3.4.3. Myocardial Infarct Size

**Figure 7:**
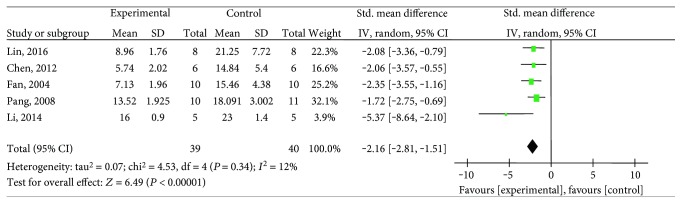
The forest plot: effects of salvianolic acid for decreasing myocardial infarct size compared with the control group.

**Figure 8:**
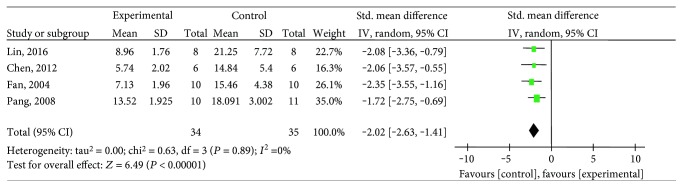
The forest plot: effects of salvianolic acid B for decreasing myocardial infarct size compared with the control group.

**Figure 9:**
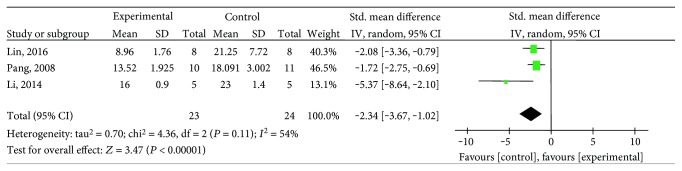
The forest plot: effects of salvianolic acid in male animals for decreasing myocardial infarct size compared with the control group.

Eight studies [25–27, 30, 31, 34, 35, 37] utilized myocardial infarct size as outcome measure. The meta-analysis of five studies [25, 27, 34, 35, 37] showed significant effects of salvianolic acid for decreasing myocardial infarct size compared with the control group (*n* = 79, SMD −2.16, 95% CI:−2.81∼−1.51, *P* < 0.00001; heterogeneity *χ*2 = 4.53, *P* = 0.34, *I*^2^ = 12%), Figure 7. After removing one salvianolic acid A study [27], the meta-analysis of four salvianolic acid B studies [25, 34, 35, 37] showed significantly decreasing myocardial infarct size compared with the control group (*n* = 69, SMD −2.02, 95% CI:−2.63∼−1.41, *P* < 0.00001; heterogeneity *χ*2 = 0.63, *P* = 0.89, *I*^2^ = 0%), Figure 8. After removing two studies [34, 35] that used female animals, the meta-analysis of three studies [25, 27, 37] showed significantly decreasing myocardial infarct size compared with the control group (*n* = 47, SMD −2.34, 95% CI:−3.67∼−1.02, *P* = 0.0005; heterogeneity *χ*2 = 4.36, *P* = 0.11, *I*^2^ = 54%), Figure 9. The reason of the high heterogeneity was possibly the use of different types of salvianolic acid. The remaining three studies [26, 30, 31] failed to pool the analysis because of the absence of primary data, but all of them reported the significant effects of salvianolic acid for decreasing myocardial infarct size compared with the control group (*P* < 0.05 or *P* < 0.01).

## 4. Discussion

### 4.1. Summary of Evidences

To our knowledge, this is the first systematic review to estimate the effects of salvianolic acid for experimental MI. Fourteen studies involving 226 animals were identified. The present study showed that salvianolic acid can reduce myocardial infarct size and promote the expression of VEGF and BVD in animal model experiments of MI, suggesting that salvianolic acid has cardioprotective function through promoting angiogenesis in the animal model of MI.

### 4.2. Limitations

First, we only searched English and Chinese databases, which may result in selective bias to some degrees [39]. Second, there are no negative studies, which may overestimate the effect to a certain degree [40]. Third, the methodological quality of included studies is generally poor, which ranges from 3 to 6. No study adopted blinded induction of model and blinded assessment of outcome. The poor methodological quality will be an inherent limitation of this systematic review, affecting the accuracy of the results [41]. MI generally occurs in patients associated with medical problems such as older age, diabetes, hypertension, and hyperlipidemia [42]. However, none of the studies adopted these appropriate animal models. Thus, we should treat present positive results cautiously because of the methodological flaws in some included studies. Finally, another weakness that should not be ignored is the heart protection of estrogen. There are two included studies [34, 35] adopting female animals. Although the specific mechanism remains unclear, the heart protection of estrogen has been observed both in clinical and in preclinical studies [43]. The sex of animals should be taken into account when designing animal experiment.

### 4.3. Implications

Angiogenesis is known as the process by which this plexus differentiates and gradually comes into being a network of functional capillaries. This step essentially is involved with the budding off of endothelial cells from the ends and lateral walls of the preexisting primitive endothelial tubules, resulting in the formation of a network of narrow capillary microvessels [44]. Clinical trials has showed that therapeutic angiogenesis make end-stage coronary artery disease patients to acquire improvements in exercise time and in symptoms of angina, as well as perfusion and left ventricular function [45]. Currently, therapeutic angiogenesis, as a new treatment of MI, has been a widespread recognition and affirmation [16–18]. This systematic review has showed that salvianolic acid can exert cardioprotection through promoting angiogenesis, making it as a potential candidate for clinical MI trials in the future.

There is no doubt that study quality is an important effect factor [41]. We suggest that further design of the studies carried out should refer to the ten-item scale [38] such as random allocation, blinded induction of model, blinded assessment of outcome, and use of anesthetic without significant intrinsic vascular protection activity. In addition, we should include appropriate animals because an unsuitable animal model may affect the validity of the experiments [46]. Myocardial infarction generally occurs in elderly patients with hypertension or hyperlipidemia [42], so using appropriate models can increase the accuracy of the results.

### 4.4. Conclusion

The salvianolic acid including salvianolic acid A, salvianolic acid B, and a mixture of salvianolic acids can reduce myocardial infarct size and promote the expression of VEGF and BVD in animal model experiments of MI, suggesting that salvianolic acid has cardioprotective function through promoting angiogenesis in the animal model of MI. However, the positive conclusion should be treated cautiously because of the methodological flaws.

## Figures and Tables

**Table 1:** Characteristics of the 15 included studies.

Study (years)	Species (sex, *n* = experimental/control group)	Weight	Model (method)	Time drug given	Anesthetic	Treatment group and administration methods	Control group	Angiogenesis outcome index	Intergroup differences (time)	Secondary outcome	Intergroup differences	Side effect
Chao et al. [25]	Male Sprague Dawley rats (8/8)	200–220 g	AMI	1 day after the surgery	Chloral hydrate [0.3 g/kg, (i.p.)]	Salvianolic acid B 24 mg/kg·4 h, i.v.	Normal saline	(1) VEGF	(1) *P* < 0.001; 8 h	(1) Myocardial infarct size	(1) *P* < 0.001; 8 h	No report

He et al. [26]	Male Sprague Dawley rats (15/15)	200–220 g	AMI	24 h after the surgery	Urethane [1.2 g/kg, intraperitoneally (i.p.)]	Salvianolic acid B 100 mg/kg·d, i.g.	Normal saline	(1) VEGF	(1) *P* < 0.05; 4 w	(1) Myocardial infarct size	(1) *P* < 0.01; 4 w	No report
								(2) BVD	(2) *P* < 0.05; 4 w	(2) LVSP	(2) *P* < 0.01; 4 w	
										(3) LVEDP	(3) *P* < 0.01; 4 w	
										(4) LVEF	(4) *P* < 0.05; 4 w	

Li et al. [27]	Male Sprague Dawley rats (5/5)	200–220 g	AMI	24 hours after the surgery	3.5% hydrochloride (3.5 mg/100 g, i.p.)	10 mg/kg/d salvianolic acid A i.v.	Normal saline	(1) BVD	(1) *P* < 0.05; 7 d	(1) Myocardial infarct size	(1) *P* < 0.01; 1 w	No report
								(2) VEGF	(2) *P* < 0.001; 7 d			

Yang [28]	Male Sprague Dawley rats (8/8)	180–200 g	AMI	1 week after the surgery	Urethane 1.2 g/kg, i.p.	Salvia extract (salvianolic acid B 20.6%; Danshensu 23.6%; protocatechualdehyde 3.9%) 100 mg/kg/d, i.g.	Normal saline	(1) VEGF	(1) *P* < 0.05; 60 d	(1) LVSP	(1) *P* < 0.05; 60 d	No report
								(2) BVD	(2) *P* < 0.05; 60 d	(2) LVEDP	(2) *P* < 0.05; 60 d	
										(3) IL	(3) *P* < 0.05; 60 d	

Fang [29]	Male Sprague Dawley rats (13/13)	220–260 g	AMI	3 days after the surgery	10% chloral hydrate (4 ml/kg, i.p.)	Salvianolate (main composition salvianolic acid B) 30 mg/kg·d, i.p.	Normal saline	(1) BVD	(1) *P* < 0.01; 4 w	No	No	No report
								(2) VEGF	(2) *P* < 0.01; 4 w			

Ma [30]	Male Sprague Dawley rats (10/10)	Three months Age	AMI	No report	3% pentobarbital 45 mg/kg, i.p.	Salvianolic acid B 1 g/(kg·d), i.g.	Normal saline	(1) BVD	(1) *P* < 0.01; 14 d	(1) Myocardial infarct size	(1) *P* < 0.01; 14 d	No report

Li [31]	Male Sprague Dawley rats (5/5)	200–220 g	AMI	1 day after the surgery	3.5% chloral hydrate (35 g/kg, i.p.)	Salvianolic acid A 10 mg/kg, i.v.	Normal saline	(1) BVD	(1) *P* < 0.001; 1 w	(1) Myocardial infarct size	(1) *P* < 0.01; 1 w	No report
								(2) VEGF	(2) *P* < 0.001; 1 w	(2) SDF-1	(2) *P* < 0.01; 1 w	
										(3) MMP-9	(3) *P* < 0.01; 1 w	

Wang [32]	Male piglets (6/6)	28 ± 10 kg	AMI	1 day after the surgery	Ketamine, 20 mg/kg, and diazepam, 0.05 mg/kg, i.m.	Salvianolate (main composition salvianolic acid B) 400 mg, i.v.	Normal saline	(1) BVD	(1) *P* < 0.01; 4 w	(1) LVEF	(1) *P* < 0.05; 4 w	No report

Nuan-Liu [33]	Male Sprague Dawley rats (8/8)	200–240 g	AMI	2 days after the surgery	10% chloral hydrate i.p.	Salvia extract (main composition salvianolic acid B) 40 mg/kg·d, i.g.	Normal saline	(1) VEGF	(1) *P* < 0.01; 4 w	No	No	No report
								(2) BVD	(2) *P* < 0.01; 4 w			

Chen [34]	Male/female Sprague Dawley rats (8/8)	180–220 g	AMI	1 day after the surgery	3% pentobarbital 30 mg/kg i.p.	Salvianolic acid B 6.4 mg, i.v.	Normal saline	(1) VEGF	(1) *P* < 0.01; 2 w	(1) Myocardial infarct size	(1) *P* < 0.01; 2 w	No report
								(2) BVD	(2) *P* < 0.01; 2 w	(2) NO	(2) *P* < 0.05; 2 w	
										(3) NOS	(3) *P* < 0.05; 2 w	

Fang [35]	Male/female Wistar rats (10/10)	250 ± 50 g	AMI	6 days before the surgery	3% pentobarbital 30 mg/kg, i.p.	Salvianolic acid B 100 mg/(kg·d), i.g.	Normal saline	(1) BVD	(1) *P* < 0.05; 6 d	(1) Myocardial infarct size	(1) *P* < 0.01; 6 d	No report
										(2) Fibroblast	(2) *P* < 0.01; 6 d	

Ye [36]	Male Sprague Dawley rats (6/7)	240 ± 60 g	AMI	48 h after the surgery	Ether inhaler	Salvia extract (main composition salvianolic acid B (40 mg/kg·d), i.g.	Normal saline1	(1) VEGF	(1) *P* < 0.01; 8 w	No	No	No report

Pang [37]	Male Sprague Dawley rats (10/11)	180–220 g	AMI	24 h after the surgery	Ether inhaler	Salvianolic acid B 120 mg/(kg·d) i.g	Normal saline	(1) BVD	(1) *P* < 0.05; 2 w	(1) Myocardial infarct size	(1) *P* < 0.05; 2 w	No report
										(2) Ventricle thickness	(2) *P* < 0.05; 2 w	

Guo et al. [38]	Female SD rats (no report)	No report	AMI	No report	No report	80 *μ*l of phosphate-buffered saline (PBS) alone and 5 × 10^6^ salvianolic acid B pretreated MSC/d	80 *μ*l of phosphate-buffered saline alone and 5 × 10^6^ MSC/d	(1) BVD	(1) *P* < 0.01; 4 w	No report	No report	No report
								(2) VEGF	(2) *P* < 0.05; 4 w			

Note: LAD: the left anterior descending coronary artery; VEGF: vascular endothelium growth factor; LVSP: left ventricular systolic pressure; LVEDP: left ventricular end-diastolic pressure; IL: infarct length; LVEF: left ventricular ejection fraction.

**Table 2:** Risk of bias of the included studies.

Study	A	B	C	D	E	F	G	H	I	J	Total
Lin et al. [25]	**√**	**√**	**√**			**√**			**√**	**√**	**6**
He et al. [26]	**√**	**√**	**√**			**√**			**√**		**5**
Li et al. [27]	**√**	**√**	**√**			**√**			**√**		**5**
Yang [28]	**√**		**√**			**√**				**√**	**4**
Fang [29]	**√**	**√**	**√**			**√**					**4**
Ma [30]	**√**		**√**			**√**				**√**	**4**
Li [31]	**√**		**√**			**√**				**√**	**4**
Wang [32]	**√**		**√**			**√**					**3**
Liu [33]	**√**		**√**			**√**					**3**
Chen [34]	**√**		**√**			**√**					**3**
Fan [35]	**√**		**√**			**√**					**3**
Ye [36]	**√**		**√**			**√**					**3**
Pang [37]	**√**		**√**			**√**					**3**
Guo et al. [38]	**√**		**√**							**√**	**3**

Note: Studies fulfilling the criteria of: A: peer reviewed publication; B: control of temperature; C: random allocation to treatment or control; D: blinded induction of model; E: blinded assessment of outcome; F: use of anesthetic without significant intrinsic vascular protection activity; G: appropriate animal model (aged, diabetic, or hypertensive); H: sample size calculation; I: compliance with animal welfare regulations; J: statement of potential conflict of interests.

## References

[1] Lauer M. S. (2012). Advancing cardiovascular research. *Chest*.

[2] Organization WH World Health Organization report. http://www.who.int/mediacentre/factsheets/fs310/zh/.

[3] Luo K. Q., Long H. B., Xu B. C. (2015). Reduced apoptosis after acute myocardial infarction by simvastatin. *Cell Biochemistry and Biophysics*.

[4] Hung J., Teng T. H., Finn J. (2013). Trends from 1996 to 2007 in incidence and mortality outcomes of heart failure after acute myocardial infarction: a population-based study of 20,812 patients with first acute myocardial infarction in Western Australia. *Journal of the American Heart Association*.

[5] Mendis S., Thygesen K., Kuulasmaa K. (2011). World Health Organization definition of myocardial infarction: 2008-09 revision. *International Journal of Epidemiology*.

[6] Dalen J. E., Alpert J. S., Goldberg R. J., Weinstein R. S. (2014). The epidemic of the 20^th^ century: coronary heart disease. *The American Journal of Medicine*.

[7] Patel N. R., Patel D. V., Murumkar P. R., Yadav M. R. (2016). Contemporary developments in the discovery of selective factor Xa inhibitors: a review. *European Journal of Medicinal Chemistry*.

[8] Antithrombotic Trialists’ (ATT) Collaboration, Baigent C., Blackwell L. (2009). Aspirin in the primary and secondary prevention of vascular disease: collaborative meta-analysis of individual participant data from randomised trials. *Lancet*.

[9] Jackson L. R., Ju C., Zettler M. (2015). Outcomes of patients with acute myocardial infarction undergoing percutaneous coronary intervention receiving an oral anticoagulant and dual antiplatelet therapy: a comparison of clopidogrel versus prasugrel from the TRANSLATE-ACS study. *JACC. Cardiovascular Interventions*.

[10] Kones R. (2011). Primary prevention of coronary heart disease: integration of new data, evolving views, revised goals, and role of rosuvastatin in management. A comprehensive survey. *Drug Design, Development and Therapy*.

[11] Krumholz H. M., Normand S. L., Wang Y. (2014). Trends in hospitalizations and outcomes for acute cardiovascular disease and stroke, 1999-2011. *Circulation*.

[12] Thompson P. D., Panza G., Zaleski A., Taylor B. (2016). Statin-associated side effects. *Journal of the American College of Cardiology*.

[13] Bledzka K., Smyth S. S., Plow E. F. (2013). Integrin alphaIIbbeta3: from discovery to efficacious therapeutic target. *Circulation Research*.

[14] Scharbert G., Wetzel L., Schrottmaier W. C., Kral J. B., Weber T., Assinger A. (2015). Comparison of patient intake of ticagrelor, prasugrel, or clopidogrel on restoring platelet function by donor platelets. *Transfusion*.

[15] Bangalore S., Guo Y., Samadashvili Z., Blecker S., Xu J., Hannan E. L. (2015). Everolimus-eluting stents or bypass surgery for multivessel coronary disease. *The New England Journal of Medicine*.

[16] Anisimov A., Tvorogov D., Alitalo A., Leppanen V. M., An Y., Han E. C. (2013). Vascular endothelial growth factor-angiopoietin chimera with improved properties for therapeutic angiogenesis. *Circulation*.

[17] Albrecht-Schgoer K., Schgoer W., Holfeld J., Theurl M., Wiedemann D., Steger C. (2012). The angiogenic factor secretoneurin induces coronary angiogenesis in a model of myocardial infarction by stimulation of vascular endothelial growth factor signaling in endothelial cells. *Circulation*.

[18] Banquet S., Gomez E., Nicol L. (2011). Arteriogenic therapy by intramyocardial sustained delivery of a novel growth factor combination prevents chronic heart failure. *Circulation*.

[19] Jiang B., Chen J., Xu L. (2010). Salvianolic acid B functioned as a competitive inhibitor of matrix metalloproteinase-9 and efficiently prevented cardiac remodeling. *BMC Pharmacology*.

[20] Joe Y., Zheng M., Kim H. J. (2012). Salvianolic acid B exerts vasoprotective effects through the modulation of heme oxygenase-1 and arginase activities. *The Journal of Pharmacology and Experimental Therapeutics*.

[21] Ho J. H., Hong C. Y. (2011). Salvianolic acids: small compounds with multiple mechanisms for cardiovascular protection. *Journal of Biomedical Science*.

[22] Fan T. P., Yeh J. C., Leung K. W., Yue P. Y., Wong R. N. (2006). Angiogenesis: from plants to blood vessels. *Trends in Pharmacological Sciences*.

[23] Murphy S. P., Murphy A. N. (2010). Pre-clinical systematic review. *Journal of Neurochemistry*.

[24] Macleod M. R., O’Collins T., Howells D. W., Donnan G. A. (2004). Pooling of animal experimental data reveals influence of study design and publication bias. *Stroke*.

[25] Lin C., Liu Z., Lu Y. (2016). Cardioprotective effect of Salvianolic acid B on acute myocardial infarction by promoting autophagy and neovascularization and inhibiting apoptosis. *The Journal of Pharmacy and Pharmacology*.

[26] He H., Shi M., Yang X., Zeng X., Wu L., Li L. (2008). Comparison of cardioprotective effects using salvianolic acid B and benazepril for the treatment of chronic myocardial infarction in rats. *Naunyn-Schmiedeberg’s Archives of Pharmacology*.

[27] Li Y. J., Duan C. L., Liu J. X. (2014). Salvianolic acid A promotes the acceleration of neovascularization in the ischemic rat myocardium and the functions of endothelial progenitor cells. *Journal of Ethnopharmacology*.

[28] Yang X.-Z. (2007). *Therapeutic Effects and Mechanisms of Danshen on Large Myocardial Infarction in Rats*.

[29] Fang L.-Y. (2015). Role of salvianolate in angiogenesis after acute myocardial infarction in rats. *International Journal of Cardiovascular Diseases*.

[30] Ma Y.-Y. (2006). *The Effects of Salidrosid and Salvianolic Acid B on Prnliferation,Migration and Apoptosis of Human Endothelial Progenitor Cells*.

[31] Li Y.-J. (2010). *Explorations on the Material Basis of pro-Angiogenic Actions of Shuangshentongguan and Underlying Mechanisms*.

[32] Wang M.-W. (2008). *Promotion of Heart Function and Collateral Blood Vessel of Salvianolate on Acute Myocardial Infarction in Swines*.

[33] Liu N., Yang L., Mao B.-y., Xu G.-c., Ye S.-s. (2015). Salvia extract promotes angiogenesis of myocardium in rats with myocardial infarction. *Chinese Journal of Pathophysiology*.

[34] Chen H.-X. (2012). Effect of salvianolic acid B on angiogenesis of ischemic myocardium in myocardial ischemia rats. *Chinese Journal of Experimental Traditional Medical Formulae*.

[35] Fang Y.-C., Fan Y. C., Zhao G. F., Zhang W. Z. (2004). Influence of salvianolic acid B on pathomorphology in myocardial infarction tats. *Journal of Tianjin University of Traditional Chinese Medicine*.

[36] Ye S.-S. (2014). Effect of salvia extract on the expression levels of VEGF in rats with myocardial infarction. *Lishizhen Medicine and Materia Medica Research*.

[37] Pang X.-L. (2008). The intervention effect of TCM on acute myocardial infarction in rats. *Tianjin Journal of Traditional Chinese Medicine*.

[38] Guo H. D., Cui G. H., Tian J. X. (2014). Transplantation of salvianolic acid B pretreated mesenchymal stem cells improves cardiac function in rats with myocardial infarction through angiogenesis and paracrine mechanisms. *International Journal of Cardiology*.

[39] Guyatt G. H., Oxman A. D., Montori V. (2011). GRADE guidelines: 5. Rating the quality of evidence—publication bias. *Journal of Clinical Epidemiology*.

[40] Franco A., Malhotra N., Simonovits G. (2014). Social science. Publication bias in the social sciences: unlocking the file drawer. *Science*.

[41] Landis S. C., Amara S. G., Asadullah K. (2012). A call for transparent reporting to optimize the predictive value of preclinical research. *Nature*.

[42] Blankstein R., Ahmed W., Bamberg F. (2012). Comparison of exercise treadmill testing with cardiac computed tomography angiography among patients presenting to the emergency room with chest pain: the Rule Out Myocardial Infarction Using Computer-Assisted Tomography (ROMICAT) study. *Circulation. Cardiovascular Imaging*.

[43] van Hout G. P., Jansen of Lorkeers S. J., Wever K. E. (2016). Translational failure of anti-inflammatory compounds for myocardial infarction: a meta-analysis of large animal models. *Cardiovascular Research*.

[44] Silvestre J. S., Smadja D. M., Lévy B. I. (2013). Postischemic revascularization: from cellular and molecular mechanisms to clinical applications. *Physiological Reviews*.

[45] Freedman S. B., Isner J. M. (2001). Therapeutic angiogenesis for ischemic cardiovascular disease. *Journal of Molecular and Cellular Cardiology*.

[46] Crossley N. A., Sena E., Goehler J. (2008). Empirical evidence of bias in the design of experimental stroke studies: a metaepidemiologic approach. *Stroke*.

